# Dynamics of protozoal excretion in the faeces of calves during the first 28 days after arrival at the fattening farm indicate infection before regrouping and show poor temporal correlation with diarrhoea

**DOI:** 10.1186/s13071-023-05911-0

**Published:** 2023-09-27

**Authors:** Jessica Bauer, Martin Kaske, Andreas Oehm, Manuela Schnyder

**Affiliations:** 1 Institute of Parasitology, Vetsuisse Faculty, University of Zurich, Winterthurerstrasse 266A, 8057 Zurich, Switzerland; 2 Swiss Calf Health Service, University of Zurich, Winterthurerstrasse 260, 8057 Zurich, Switzerland

**Keywords:** Fattening calves, Group rearrangement, Intestinal protozoa, *Eimeria*, *Giardia*, *Cryptosporidium*, Excretion pattern

## Abstract

**Background:**

Calves in dairy cattle production in Switzerland are transported to a fattening farm at the age of 3–5 weeks, and frequently suffer from diarrhoea within the first 14 days after arrival. To characterise the role of intestinal protozoa in this, we investigated the excretion dynamics of *Eimeria*, *Cryptosporidium* and *Giardia* during the first 28 days after the arrival and regrouping of calves at fattening farms.

**Methods:**

A total of 610 faecal samples from 122 calves (mean age 37.3 days; mean body weight 79.8 kg) were collected on seven different fattening farms during the first 28 days after the arrival and regrouping of the animals. The farms were visited between January and April (cold season; *n* = 4) and between June and August (warm season; *n* = 3). The samples were collected rectally on days 1, 4, 7, 14 and 28, assessed for consistency, and analysed using the McMaster method for quantitative determination of the number of *Eimeria* oocysts per gram of faeces (OPG), flotation for morphological differentiation of the unsporulated *Eimeria* oocysts, a concentration method for the semi-quantitative determination of *Giardia* cysts, and modified Ziehl–Neelsen staining for semi-quantitative determination of *Cryptosporidium* oocysts.

**Results:**

Overall, 50.8% (62/122) of the animals had diarrhoea during the study period. However, the faecal excretion of protozoal pathogens was neither associated with diarrhoea nor with body weight gain of the animals. Altogether, 90.2% (110/122) of the calves were *Eimeria* positive. *Eimeria zuernii* was excreted by 51 (41.8%) and *Eimeria bovis* by 68 (55.7%) animals. In the warm season more animals tested positive for *Eimeria* and OPGs were higher than in the cold season. There was no correlation between the age of the calves and the OPG values. Overall, 64.8% (79/122) of the calves excreted *Eimeria* oocysts within the first 7 days, indicating that they had been infected with the parasite on the dairy farm of origin. Eighty-nine calves (73.0%) excreted *Giardia* cysts, with more positive animals in the cold (80.3%) compared with the warm season (64.3%). Only *Giardia duodenalis* assemblage E was identified. *Cryptosporidium* oocysts were microscopically detected in 14 animals (11.5%) on five farms. *Cryptosporidium* spp. were present in a total of 12 animals, i.e. *Cryptosporidium parvum* in nine, *Cryptosporidium ryanae* in two, and *Cryptosporidium bovis* in one animal.

**Conclusions:**

A better understanding of the temporal dynamics of protozoal infections in calves is helpful for the implementation of appropriate measures to protect the health of these animals at a critical phase in their lives. Our results indicate that factors other than those examined in the present study contributed to the onset of diarrhoea in the calves.

**Graphical abstract:**

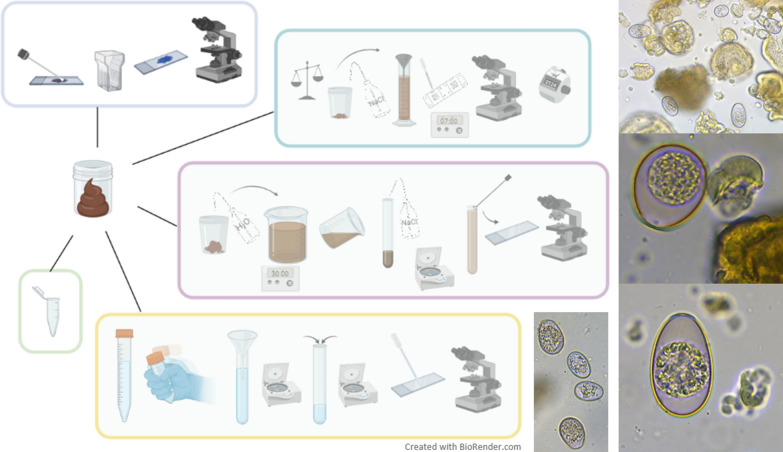

**Supplementary Information:**

The online version contains supplementary material available at 10.1186/s13071-023-05911-0.

## Background

Gastrointestinal signs, together with respiratory ones, are among the most frequent clinical signs detected in calves after their arrival at fattening farms in Switzerland [1–3]. In the case of diarrhoea, the aetiology is often multifactorial and may include both infectious and non-infectious components [4]. Diarrhoeal diseases not only affect the well-being of the calves, but also lead to notable economic losses due to the cost of veterinary treatment and additional care, lower body weight gain, reduced quality of the carcass, and higher mortality [5–7].

The occurrence of different causative pathogens depends, among other factors, on the age of the animal [4, 8–10]. In Switzerland, calves with a minimum age of 21 days are transported to fattening facilities. The parasitic pathogens that play a major role in diarrhoeal diseases in this age group are *Eimeria* spp., *Giardia duodenalis* and *Cryptosporidium parvum* [11, 12].

Under conventional farming conditions, cattle frequently harbour *Eimeria*. In calves from 3 weeks to 12 months of age, *Eimeria* infections cause intestinal lesions, leading to clinical signs such as watery and haemorrhagic diarrhoea, anorexia, dehydration, and body weight loss [13, 14]. In previous studies, *Eimeria* herd prevalence in calves varied between 86 and 100%, while infection rates in individual animals ranged from 33 to 83.7% [15–19]. Studies from Switzerland indicated *Eimeria* spp. excretion rates ranging from 28.0 to 43.0% [20–22]. Due to the presence of apathogenic species of *Eimeria*, species-specific differentiation in this genus is relevant [23]: of the 13 *Eimeria* species that infect cattle, *Eimeria zuernii*, *Eimeria bovis* and *Eimeria alabamensis* are considered the most pathogenic [14, 24–26]. *Eimeria alabamensis* is the predominant species in grazing cattle [26–28]. Following infection, calves develop species-specific immunity [14]. Immunologically naive or immunocompromised animals, as well as animals suffering from stress, or other diseases, are at particular risk of eimeriosis [29].

The pathogenesis of *G. duodenalis* in livestock is not clearly understood. Based on genetic analyses, eight genotypes (assemblages A–H) have been defined, of which assemblages A and E occur in calves [30]. Infections with these assemblages can cause diarrhoea and a reduction in body weight gain, although subclinical infections are thought to be more likely [31, 32]. Previous studies indicated a notable variation in infection rates. For instance, Geurden et al. [31] examined faecal samples from calves with an average age of 7.8 weeks from four different European countries: the overall apparent prevalence of *G. duodenalis* was 45.4%. The infection rates within the individual countries were as follows: 32.2% in Italy, 39.6% in France, 51.2% in Germany and 54.9% in the United Kingdom. In Norway, the infection rate was 49.0% [33].

Infection with *C. parvum* is a major problem in calves shortly after they are born [9]. *Cryptosporidium parvum*, *Cryptosporidium bovis*, *Cryptosporidium ryanae*, and *Cryptosporidium andersoni* are commonly found in cattle, but only *C. parvum* has been associated with clinical disease. Depending on the country of origin and age of the animals, reported infection rates varied between 30.0 and 47.9% [19, 22, 34, 35]. Older calves (> 6 weeks) are often asymptomatic and frequently excrete apathogenic species [36].

Both *Cryptosporidium* oocysts and *Giardia* cysts are infective when they are excreted; their high tenacity [37] has an effect on both their dissemination and transmission. Moreover, *G. duodenalis* and *C. parvum* are of public health significance as they are potential zoonotic pathogens [38–40].

Moving calves from their farm of origin to the fattening farm represents a critical phase for the animals. The immune system of the animals has not yet fully matured at this point due to their young age. Also, transport and regrouping of animals from different farms represent important stressors [41–43]. Knowledge of the excretion patterns of intestinal protozoa in calves is helpful for a better understanding of the dynamics of infections of these animals with protozoal pathogens of relevance, and for the design of appropriate preventive measures for implementation at the best time to maintain and improve the health of calves at a critical phase in their lives.

The objective of this study was to examine calves for protozoal diarrhoeic pathogens for the 28 days following their arrival at fattening farms, to investigate possible correlations between protozoal excretion and the occurrence of diarrhoea and changes in body weight gain.

## Methods

### Study population and faecal sampling

Based on the motivation of the farmers to participate in this study, seven fattening farms were selected by the Swiss health service for calves [Schweizer Kälbergesundheitsdienst (KGD); www.kgd-ssv.ch]. The following requirements had to be fulfilled: no history of clinical coccidiosis on the farms, no preventive anti-coccidial treatments given on the farms, calves grouped from different farms of origin until mature enough for slaughter, and an all-in-all-out management system. The farms were visited at different time points between July 2019 and April 2022: three farms were visited between June and August (warm season) and four farms between January and April (cold season). The calves were housed indoors, on deep straw, in groups, and fed with milk replacer. They had ad libitum access to water and hay.

The size of the groups ranged from 12 to 40 animals. The farms were visited after the arrival of the calves at the fattening farm (day 0) on days 1, 4, 7, 14 and 28 to analyse the dynamics of protozoal excretion. In principle, a minimum of 20 calves per farm were examined. On farms where less than 20 calves were present, all of the calves were included in the study. A total of 122 crossbred calves aged 19–65 days at the start of the study (mean, 37.3; SD, 10.3) were included. Most of the calves were male (86.9%, 106/122), and the number of calves sampled during the warm (*n* = 56) and cold season (*n* = 66) was comparable. The data of six calves were excluded due to their unexpected death (*n* = 4), individual treatment for diagnosed coccidiosis (*n* = 1), or incomplete results over the whole study period (*n* = 1). Individual faecal samples were taken rectally from each calf. At the same time, a short examination was carried out to determine the general condition of the calves (categorised into reduced, physiological, good, very good), the condition of their heart and lungs through auscultation (physiological condition, increased vesicular sound, or pathological murmurs), and to check the umbilicus (on day 1; physiological condition, abnormalities). The body weight of the calves was determined on days 1 and 28 by use of a measuring tape [44]. Faecal consistency was recorded as normal, pasty, semi-liquid, or watery. Diarrhoea was defined as semi-liquid or watery faeces [45, 46].

### Faecal analyses

Upon arrival at the laboratory, the faecal samples were stored at 4 °C, then analysed within 1 day; 1–2 g of faeces was stored in a 1.5-ml Eppendorf tube at − 20 °C for further investigation.

### Microscopic examination

The McMaster method, with a sensitivity of 50 oocysts, was applied to determine the number of *Eimeria* oocysts per gram of faeces (OPG) [47]. Positive samples were further assessed using a sedimentation/flotation method [47]. From each positive sample, 50 non-sporulated *Eimeria* oocysts were microscopically examined (400× magnification), and the pathogenic species *E. zuernii* and *E. bovis* were morphologically differentiated based on the presence or absence of a micropyle and the size and the shape of the oocysts [48]. All other oocysts were not differentiated further than genus level; all of them were determined to be *Eimeria* spp. For both methods, saturated NaCl (density 1.20 g/mL) was used as the flotation solution.

The sodium acetate-acetic acid-formalin concentration method was used for the semi-quantitative examination of faecal samples for *Giardia* cysts [47]. The samples were evaluated under a microscope at 400× magnification and classified semi-quantitatively as follows: one plus (+) when at least one *Giardia* cyst was present in the sample; ++ when more than two, but fewer than 10 *Giardia* cysts were identified in several fields of view; and +++ when there were more than 10 *Giardia* cysts in several fields of view.

Faecal smears were semi-quantitatively examined for *Cryptosporidium* oocysts using the modified Ziehl–Neelsen stain [47]: the samples were evaluated under the microscope at 1000× magnification and classified as described for *Giardia* (negative, +, ++, or +++).

### DNA extraction and polymerase chain reaction

All microscopically examined samples positive for *Cryptosporidium* spp. (*n* = 24) were examined further. DNA was extracted from faecal samples stored at − 20 °C using the QIAamp DNA Stool Mini Kit (Qiagen, Hilden, Germany). First, 200 mg of faeces and 100 µl distilled H_2_O were put into a 2-ml tube and vortexed, and an alkaline lysis was performed as described in Stefanić et al. [49], after which the manufacturer’s instructions for use of the kit were followed. A 740-base pair fragment of the nuclear 18S ribosomal RNA gene was targeted and amplified by nested polymerase chain reaction (PCR) [50]. The reaction volume (50 µl) contained 25 µl Mastermix (Qiagen multiplex PCR kit), 19 μl nuclease-free water, 0.5 μl each of 100 mM oligonucleotide primers and 5 μl of genomic DNA template. For the second PCR, 0.5 μl template from the previous PCR was used. The cycling protocol for both reactions included an initial cycle at 95 °C for 15 min, followed by 40 cycles of 94 °C for 30 s, 56 °C for 60 s, 72 °C for 60, and a final extension at 72 °C for 10 min.

To further examine the microscopically positive *Giardia* spp. samples, a nested PCR was performed on 58 samples. The samples were selected as follows: all samples of *Giardia*-positive calves presenting with diarrhoea (*n* = 19), and the same number of *Giardia*-positive samples from calves without diarrhoea. A 384-base pair fragment of the β-giardin gene was targeted and amplified using the forward primer G7 (5′-AAGCCCGACGACCTCACCCGCAGTGC-3′) and the reverse primer G759 (5′GAGGCCGCCCTGGATCTTCGAGACGAC-3′) in the first reaction [51] and the forward primer 5′-GAACGAGATCGAGGTCCG-3′, and reverse primer 5′-CTCGACGAGCTTCGTGTT-3′ in the second reaction [52]. The reaction volume (25 µl) contained 12.5 µl Mastermix (Qiagen multiplex PCR kit), 10 μl nuclease free water, 0.25 μl each of 100 mM oligonucleotide primers and 2 μl of genomic DNA template. For the second PCR, 2 μl template from the previous PCR was used. The cycling protocol for both reactions included an initial cycle of 95 °C for 15 min, followed by 40 (nest 1)/35 (nest 2) cycles at 94 °C for 30 s, 65 °C (nest 1)/55 °C (nest 2) for 30 s, 72 °C for 60, and a final extension at 72 °C for 10 min. The primers were manufactured by Microsynth (Microsynth, Balgach, Switzerland), the reactions were performed in a C1000 Touch thermal cycler (Bio-Rad) and the PCR products were subjected to electrophoresis on 1.5% agarose gels and visualised with ultraviolet light. The amplicons were purified with the MinElute PCR Purification Kit (Qiagen), the DNA was quantified by using a NanoDrop One Microvolume UV–Vis Spectrophotometer (Thermo Fisher Scientific, Waltham, MA) and sequenced unidirectionally using Sanger technology by Microsynth. All obtained sequences were compared with the ones in the GenBank nucleotide database, using a Basic Local Alignment Search Tool (BLAST) search (http://www.blast.ncbi.nlm.nih.gov). *Giardia* and *Cryptosporidium* sequences were analysed by multiple alignment using Genious Prime version 2022.2.1 to identify species and sub-genotype, as described by Gillhuber et al. [53] for *Giardia*, and by using reference sequences from published sequences [50] for *Cryptosporidium* (*C. parvum*, MT611069; *C. bovis*, MT611082; *C. ryanae*, MT611089).

### Data analysis

All analyses were carried out using Microsoft Excel or R version 4.2.0 using the R Studio interface (R Project for Statistical Computing; R Foundation, R Development Core Team, Vienna). All continuous variables were assessed for normality by creating quantile–quantile plots [54] and by applying the Shapiro–Wilk test [55]. Levene’s test [56] was implemented to test for variance homogeneity. If predictors were normally distributed, univariate ANOVA was carried out for comparison among farms. For season, a* t*-test for independent samples was conducted. When predictor variables were not normally distributed, farms were compared via the Kruskal–Wallis test [57], and seasons by using the Mann–Whitney *U*-test [58]. Categorical variables were evaluated using Pearson’s chi-squared test [59]. Fisher’s exact test was applied when there were fewer than five observations within a category [60]. The Bonferroni correction was applied to adjust* P*-values for multiple comparisons [61]. To investigate the correlation of the age and number of oocysts, a Spearman’s correlation was carried out. To identify potential associations of body weight gain and the presence of diarrhoea, respectively, with a set of covariates (i.e., OPG, season, presence of *Giardia* spp./*Cryptosporidium* spp./*Eimeria* spp.), linear mixed models or binomial logistic models, respectively, were fitted. Farm and calf identity were included as random effects (calf identity nested within farm) to account for repeated measures and potential between-calf and between-farm heterogeneity. A manual stepwise backwards selection procedure was chosen to obtain the best model given the data. Initially, all predictors were included in the model and one predictor at a time was removed. Candidate models were ranked after the removal of each covariate using the compare_performance() function in the R performance package [62]. Lower Akaike information criterion and Bayesian information criterion values indicated better model fit [63]. To assess differences in OPG between seasons, a cumulative link mixed model (ordered logit model) was built at calf level by applying the clmm() function from the ordinal package [64]. OPG category (≤ 500 OPG, > 500–5000 OPG, > 5000 OPG) was the ordinal, categorical response, and farm and calf identity were included as random effects. The proportional odds assumption was tested for the model and a *P*-value of 0.4 indicated no violation, assuming a distinct order in the categories of the response variable. Season and examination date were included as fixed effects.

To assess differences in the excretion of *Giardia* cysts, mixed-effects binomial logistic regression models were built at calf level by applying the glmer() function from the lme4 package [65]. The target variable *Giardia* (negative, +, ++, +++) was transformed to a ‘positive’ or ‘negative’ factor. Farm and calf identity were included as random effects, season and examination days as fixed effects. Throughout the analyses, statistical significance was set at *P* ≤ 0.05.

## Results

Overall, 122 calves and 610 faecal samples were examined. Upon their arrival at the fattening farms, the mean age of the calves was 37.3 ± 10.3 days (minimum 19, maximum 65 days) and their body weight ranged from 63.0 to 98.0 kg (mean, 79.8 ± 6.4). No difference in body weight (ANOVA, *F* = 1.4, *P* = 0.208) or age (Kruskal–Wallis rank sum test, χ^2^ = 11.4, *P* = 0.076) was detected between the farms on the first day. On average, the calves gained 16.1 ± 10.6 kg (minimum − 6, maximum 46.0 kg), and reached a mean body weight of 95.9 ± 12.6 kg (minimum 69.0, maximum 131.0 kg) on day 28. On day 28, the body weight of the calves differed between the farms (Kruskal–Wallis rank sum test, χ^2^ = 33.5, *P* = 0.0001), but not between seasons (Wilcoxon signed-rank test, *Z* =− 1.8, *P* = 0.08). There was no association between the body weight gain of the calves and the presence of the investigated protozoan pathogens in the faecal samples. Detailed data are available in Additional file 3: Table S1 and Additional file 4: Tables S2-S4.

### *Eimeria*

**Fig. 1:**
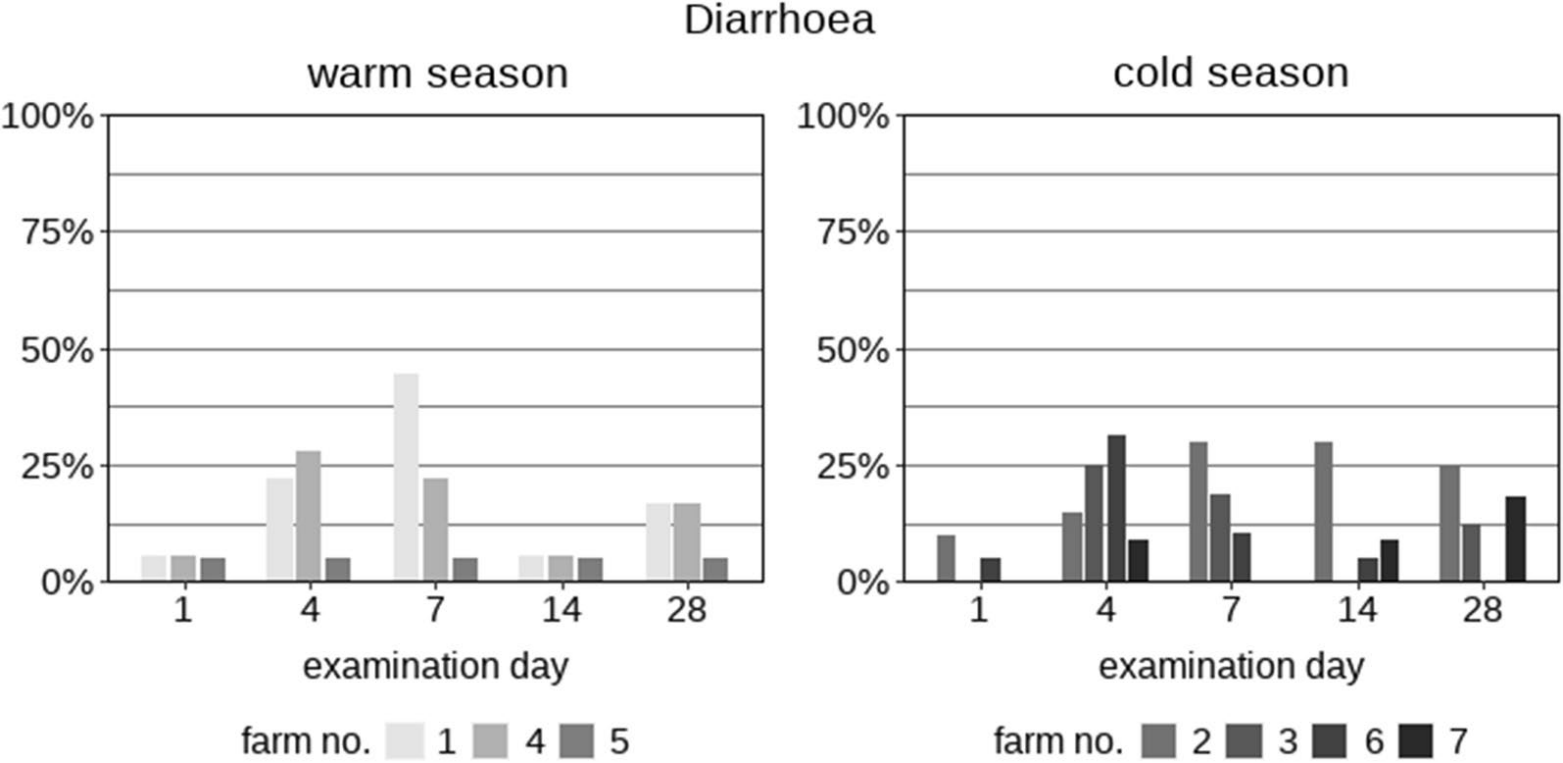
Percentage of calves with diarrhoea on seven farms sampled during the warm (*n* = 3) or cold (*n* = 4) seasons on days 1, 4, 7, 14 and 28 after arrival and grouping of the animals at the fattening farm

Over the whole study period, 90.2% (110/122) of the calves excreted *Eimeria* oocysts on at least one day. Forty-five calves (36.9%) had an OPG > 5000, a limit previously considered clinically relevant [23], at least once (Tables 1, 2). The OPG values varied between the seasons. Both the mean and median values were consistently lower in the cold season than in the warm season (Table 3). In addition, the OPG values were higher on the earlier study days (days 1, 4 and 7) than on the later study days (days 14 and 28). There was no correlation between the age of the calves and the measured OPG values for *Eimeria*. However, when the data for *E. bovis* and *E. zuernii* were examined independently, a statistically significant correlation between OPG and the age of the animals was found for *E. zuernii* on day 28 (*R* = − 0.47, *P* = 0.02); more *E. zuernii* oocysts were excreted by animals when they were younger than when tested on day 28, when they were older (Fig. 1).

**Table 1:** Coproscopic results and presence of diarrhoea in 122 calves examined on five different days (1, 4, 7, 14 and 28 days after arrival at the fattening farm) during the warm (June–August; *n* = 3 farms) and cold season (January–April; *n* = 4 farms)

	*Eimeria* positive		*Eimeria* OPG > 5000		*Giardia* positive		*Cryptosporidium* positive		Diarrhoea	
	*n*	%	*n*	%	*n*	%	*n*	%	*n*	%
Warm season										
	Farm 1 (*n* = 18)									
Day 1	13	72.2	7	38.9	2	11.1	0	0	1	5.6
Day 4	17	94.4	6	33.3	2	11.1	2	11.1	4	22.2
Day 7	14	77.8	5	27.8	4	22.2	3	16.7	8	44.4
Day 14	14	77.8	2	11.1	8	44.4	1	5.6	1	5.6
Day 28	16	88.9	4	22.2	3	16.7	0	0	3	16.7
Mean	15	82.2	5	26.7	4	21.1	1	11.1	3	18.9
	Farm 4 (*n *= 18)									
Day 1	9	50.0	1	5.6	3	16.7	0	0	1	5.6
Day 4	10	55.6	3	16.7	4	22.2	0	0	5	27.8
Day 7	10	55.6	2	11.1	3	16.7	0	0	4	22.2
Day 14	0	0	0	0	10	55.6	1	5.6	1	5.6
Day 28	15	83.3	2	11.1	6	33.3	0	0	3	16.7
Mean	9	48.9	2	8.9	5	28.9	0	1.1	3	15.6
	Farm 5 (*n* = 20)									
Day 1	11	55.0	5	25.0	3	15.0	1	5.0	1	5.0
Day 4	11	55.0	4	20.0	4	20.0	0	0	1	5.0
Day 7	13	65.0	4	20.0	6	30.0	2	10.0	1	5.0
Day 14	9	45.0	0	0	5	25.0	3	15.0	1	5.0
Day 28	16	80.0	0	0	3	15.0	0	0	1	5.0
Mean	12	60.0	3	13.0	4	21.0	1	6.0	1	5.0
Cold season										
	Farm 2 (*n* = 20)									
Day 1	8	40.0	1	5.0	11	55.0	0	0	2	10.0
Day 4	7	35.0	2	10.0	4	20.0	0	0	3	15.0
Day 7	10	50.0	1	5.0	5	25.0	0	0	6	30.0
Day 14	6	30.0	1	5.0	6	30.0	0	0	6	30.0
Day 28	6	30.0	0	0	1	5.0	0	0	5	25.0
Mean	7	37.0	1	5.0	5	27.0	0	0.0	4	22.0
	Farm 3 (*n* = 16)									
Day 1	5	31.3	1	6.3	5	31.3	1	6.3	0	0
Day 4	3	18.8	0	0	3	18.8	1	6.3	2	12.5
Day 7	4	25.0	0	0	4	25.0	1	6.3	2	12.5
Day 14	5	31.3	0	0	10	62.5	3	18.8	0	0
Day 28	12	75.0	1	6.3	8	50.0	0	0	0	0
Mean	6	36.3	0	2.5	6	37.5	1	7.5	1	5.0
	Farm 6 (*n* = 19)									
Day 1	7	36.8	1	5.3	7	36.8	0	0	1	5.3
Day 4	4	21.1	1	5.3	4	21.1	0	0	6	31.6
Day 7	8	42.1	2	10.5	3	15.8	0	0	2	10.5
Day 14	7	36.8	1	5.3	7	36.8	0	0	1	5.3
Day 28	10	52.6	2	10.5	7	36.8	0	0	0	0
Mean	7	37.9	1	7.4	6	29.5	0	0.0	2	10.5
	Farm 7 (*n* = 11)									
Day 1	5	45.5	1	9.1	6	54.5	0	0	0	0
Day	7	63.6	2	18.2	8	72.7	0	0	1	9.1
Day 7	3	27.3	0	0	4	36.4	0	0	0	0
Day 14	4	36.4	0	0	4	36.4	1	9.1	1	9.1
Day 28	5	45.5	1	9.1	5	45.5	0	0	2	18.2
Mean	5	43.7	1	7.3	5	49.1	0	1.8	1	7.3

*OPG* Oocysts per gram of faeces

**Table 2:** Details of calves diagnosed positive for *Eimeria*, specifically for *Eimeria zuernii* and *Eimeria bovis*, and for *Giardia* and *Cryptosporidium*

	Warm season (*n* = 56)		Cold season (*n* = 66)		Total (*n* = 122)	
	*n*	%	*n*	%	*n*	%
*Eimeria*						
Positive	55	98.2	55	83.3	110	90.2
> 5000 OPG	32	57.1*	13	19.7*	45	36.9
Positive before day 14	42	75.0	30	45.5	79	64.8
Positive after day 14	13	23.2	16	24.2	31	25.4
*Eimeria zuernii*						
Positive	31	55.4	20	30.3	51	41.8
> 5000 OPG	4	7.1	2	3.0	6	4.9
Positive before day 14	27	48.2	9	13.6	36	29.5
Positive after day 14	4	7.1	11	16.7	15	12.3
*Eimeria bovis*						
Positive	39	69.6	29	43.9	68	55.7
> 5000 OPG	15	26.8	1	1.5	16	13.1
Positive before day 14	31	55.4	18	27.3	49	40.2
Positive after day 14	8	14.3	11	16.7	19	15.6
*Giardia*						
Positive	36	64.3*	53	80.3*	89	73.0
*Cryptosporidium*						
Positive	10	17.9	4	6.1	14	11.5

* *P* ≤ 0.05 (significant difference between the cold and the warm seasons)

**Table 3:** Maximum (*max*.), mean, SD and median *Eimeria* OPGs of calves examined in the warm and cold seasons on sampling days 1, 4, 7, 14 and 28 after their arrival at the seven fattening farms

	Warm season (farms 1, 4, 5)			Cold season (farms 2, 3, 6, 7)			
Day	Farm 1	Farm 4	Farm 5	Farm 2	Farm 3	Farm 6	Farm 7
OPG max.							
1	30,550	20,650	33,950	11,350	5400	28,850	71,000
4	108,400	12,500	49,500	9250	2750	29,500	10,350
7	40,200	35,500	24,050	21,500	800	22,700	1850
14	15,550	0	900	5500	2700	5250	4100
28	11,150	6950	1950	4200	5650	19,400	8650
OPG mean							
1	8264	2103	4048	783	469	1626	6759
4	9506	1911	6628	793	244	1574	2059
7	4397	2939	4545	1460	122	1700	323
14	2119	0	143	525	231	418	609
28	2892	1606	465	345	813	2005	1482
OPG SD							
1	10,210	4885	8162	2544	1353	6598	21,315
4	25,078	3587	14,847	2284	722	6763	3766
7	9387	8254	7674	4805	260	5369	642
14	3931	0	257	1356	674	1282	1378
28	3381	2171	540	1006	1483	4700	2737
OPG median							
1	3400	125	250	0	0	0	0
4	1775	175	750	0	0	0	150
7	475	400	1000	25	0	0	0
14	750	0	0	0	125	0	0
28	1950	675	275	0	0	150	0

Farms are grouped according to the season in which the calves were examined

Seventy-nine calves (64.8%, *n* = 122) were positive on one of the first 3 examination days (days 1, 4, 7). In contrast, 31 (25.4%, *n* = 122) were positive for the first time on the fourth or fifth day of examination (day 14 or 28), whereas 12 (9.8%, *n* = 122) remained negative throughout the study period (Table 2). In the warm season, of the animals positive for* Eimeria*, 37.5% tested positive on 4 out of the 5 days of examination (21/56); in the cold season, 27.3% of the animals positive for this genus tested positive on 1 of the 5 days of examination (18/66) (Additional file 1: Fig. S1; Additional file 4: Tables S2-S3).

The ordinal regression model indicated a higher odds for calves to excrete a higher level of OPG during the warm season than the cold season {OR 3.7 [95% confidence interval (CI) 1.9–7.3], *P* < 0.001}. The odds were lower for the calves to excrete more OPG on day 14 compared with the first examination, on day 1 [OR 0.4 (CI 0.2–0.7), *P* = 0.001; Table 4].

**Table 4:** Ordinal regression model results for excretion by the calves of a high level of *Eimeria *OPG (where low is < 500 OPG, intermediate is 500—5000 OPG, and high is > 5000 OPG) and the covariates season and examination day

Variable	Category	Estimate	OR	CI	*P* value
Season	Warm season	Reference	–	–	–
	Cold season	1.3	3.7	1.9 –7.3	< 0.001*
Examination days	1	Reference	–	–	–
	4	− 0.02	0.98	0.6 –1.6	0.9
	7	− 0.02	0.98	0.6 –1.6	0.9
	14	− 0.9	0.4	0.2 –0.7	0.001*
	28	0.4	1.5	0.9 –2.5	0.09

Number of calves, 122; number of observations, 610

*OR* Odds ratio, *CI* 95% confidence interval

* *P* < 0.05

**Fig. 2:**
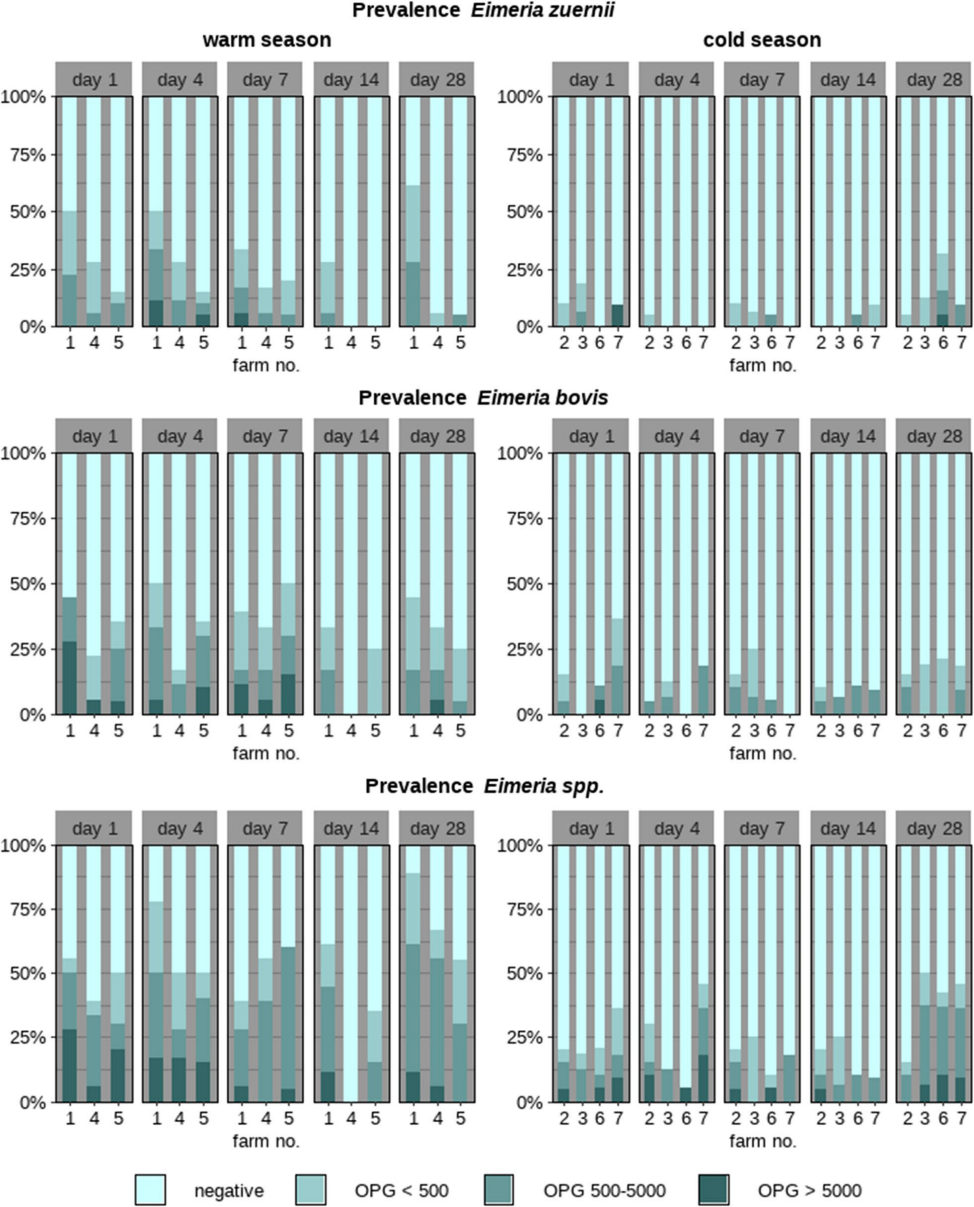
Prevalence of the pathogenic *Eimeria zuernii* and *Eimeria bovis* and the apathogenic *Eimeria* spp. in the warm (*n = *3 farms) and the cold seasons (*n* = 4 farms) on 5 examination days after arrival of the calves at the fattening farms

*Eimeria zuernii* was excreted by 51 (41.8%, *n* = 122) and *E. bovis* by 68 (55.7%, *n* = 122) animals (Table 2). In the cold season, fewer *E. zuernii*-positive and *E. bovis*-positive samples were detected compared to the warm season (Fig. 2). Adding up the relative OPG rates determined for each animal on the specific examination days showed that the relative proportion of pathogenic *Eimeria* species (*E. zuernii, E. bovis*) decreased from 39.6% on examination day 1 to 27.5% on day 28 (Additional file 2: Fig. S2).

### *Giardia*

**Fig. 3:**
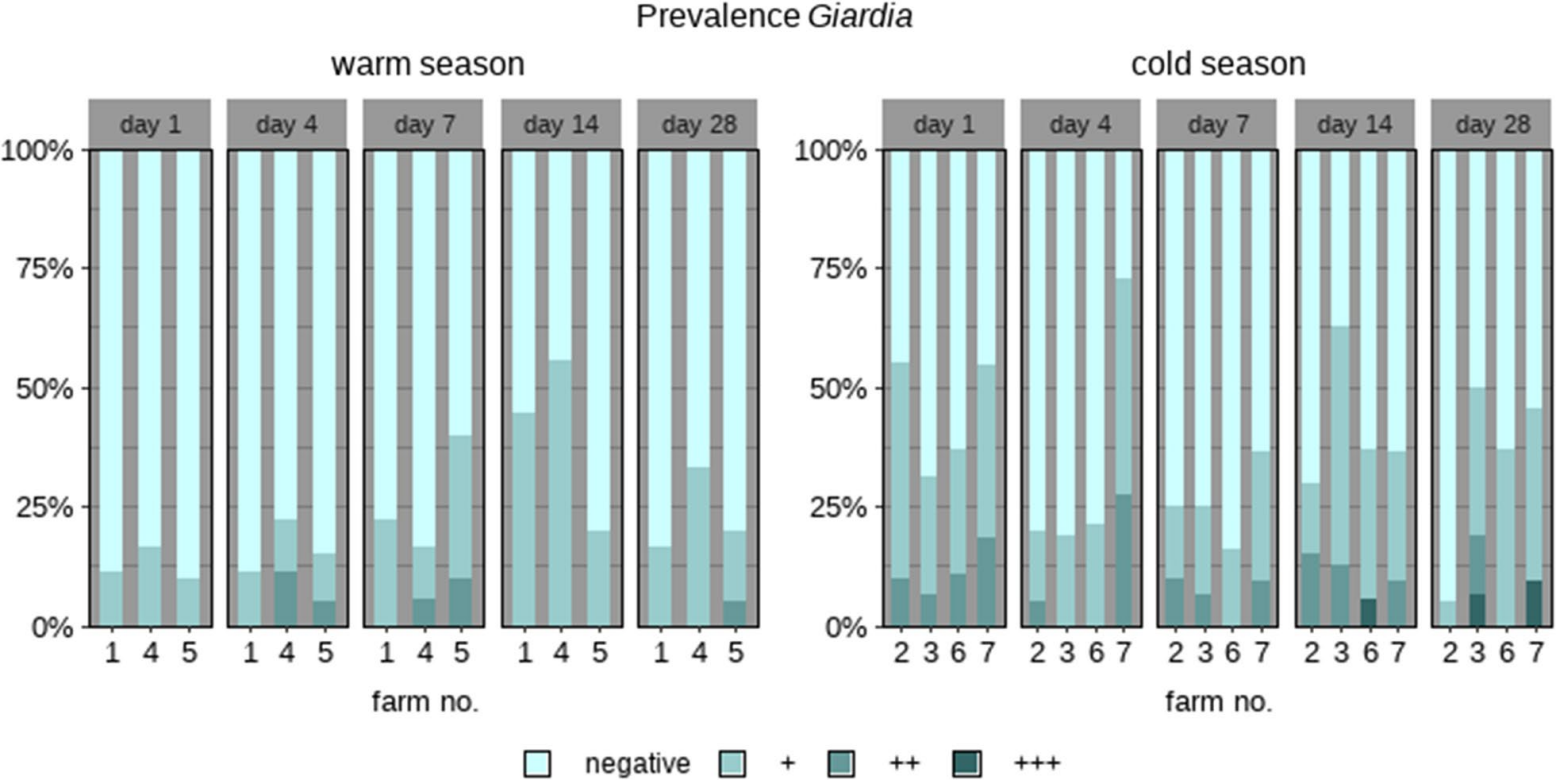
Relative proportions of *Giardia*-negative and *Giardia*-positive calves (samples divided into categories according to numbers of cysts excreted, where + is low, ++ is intermediate, and +++ is high) on seven farms sampled during the warm (*n* = 3) or the cold (*n* = 4) seasons on days 1, 4, 7, 14 and 28 after arrival and regrouping of the animals at the fattening farms

*Giardia*-positive animals were identified on all farms, with 73.0% of the animals (89/122) testing positive for this pathogen at least once during the study period; *Giardia* excretion rates varied between 11.1 and 62.5% on the farms (Tables 1 and 2). Most animals (28.7%, 35/122) excreted *Giardia* only on 1 of the 5 examination days. In the warm season, 64.3% (36/56) of the calves excreted *Giardia* cysts at least once, and in the cold season 80.3% (53/66) of the calves excreted *Giardia* cysts at least once: the odds were higher for the calves to be infected with *Giardia* in the cold season than in the warm season [OR 1.8 (CI 1.1–2.8),* P* = 0.02] (Table 5). Animals with a +++ faecal sample were only found in the cold season (3/66, 4.5%; Fig. 3). Overall, the highest rate of positive samples (49/122, 40.2%) was for calves sampled on day 14. The biggest disparity between the cold and warm season in excretion rates of *Giardia* cysts was on day 1: in the warm season, the percentage of positive animals on day 1 was 8.9% (5/56) and in the cold season 43.9% (29/66) (Fig. 3). In the warm season, the odds were higher for the calves to be positive for *Giardia* on examination days 7 and 14, and lower for day 7 in the cold season (reference, examination day 1; Table 5).

**Table 5:** Binomial regression model results for *Giardia* excretion in the warm and cold seasons, with covariates season and examination day

Variable	Category	Estimate	OR	CI	*P*-value
Season	Warm season	Reference	–	–	–
	Cold season	0.6	1.8	0.2 – 0.4	0.017*
Examination day, warm season	1	Reference	–	–	–
	4	− 0.7	1.4	0.5 – 4.2	0.6
	7	− 0.7	2.9	1.0 – 8.3	0.049*
	14	− 0.8	5.6	2.0 – 16.0	0.001*
	28	− 0.7	2.3	0.9 – 6.7	0.123
Examination day, cold season	1	Reference	–	–	–
	4	− 0.6	0.5	0.2 – 1.0	0.056
	7	− 0.6	0.4	0.2 – 0.8	0.012*
	14	− 0.6	0.9	0.4 – 1.8	0.7
	28	− 0.6	0.6	0.3 – 1.1	0.1

Number of calves, 122; number of observations, 610. For abbreviations, see Tables 1 and 4

* *P* < 0.05

Of the 38 samples that were investigated by PCR, eight (21.1%) were positive, successfully sequenced, and aligned: all samples contained *G. duodenalis* assemblage E. Of the positive animals, five had diarrhoea at the time of sampling; all five calves originated from different farms. Samples from three of the five were collected in the cold season; one sample each was obtained on examination days 1, 7 and 28, and two samples on day 14.

### *Cryptosporidium*

Over the entire study period, 14 (11.5%, *n* = 122) animals were microscopically positive for *Cryptosporidium*, i.e. 10 during the warm and four during the cold season (Table 2). Positive samples were obtained from farms 1, 3, 4, 5 and 7 on examination days 1, 4, 7 and 14 (Table 1). Most of the positive samples were obtained on day 14 (8/20). Since four animals were positive on several study days, there was a total of 20 microscopically positive faecal samples. Of these, 16 were identified as positive by PCR. The subsequent sequencing identified the following *Cryptosporidium* species: *C. parvum* (9/14), *C. ryanae* (2/14), and *C. bovis* (1/14). Two samples could not be successfully sequenced. Three animals from different farms presented with diarrhoea at the same time as the detection of *Cryptosporidium*; two of these calves were positive for *C. parvum* and one was positive for *C. ryanae*.

### Diarrhoea

The highest number of animals presenting with diarrhoea (8/18, 44.4%) was recorded on day 7 on farm 1 during the warm season (Table 1, Fig. 1). Across farms, diarrhoea most frequently appeared on day 7 (13/56) in the warm season, and on day 4 (16/66) in the cold season. However, there were no statistically significant differences between the seasons.

Overall, 50.8% (62/122) of the animals presented with diarrhoea at some point during the study. Most commonly, the calves had diarrhoea on a single study day (45/122, 36.9%). Only 15 calves (12.3%) suffered from diarrhoea on 2 examination days, one animal had diarrhoea on 3 examination days (1/122, 0.8%) and another animal had diarrhoea on all 5 examination days (1/122, 0.8%).

The statistical models did not show any relation between the occurrence of diarrhoea and the excretion of the investigated protozoa.

## Discussion

Over the study period, different trends were observed in the protozoal pathogens that can potentially cause diarrhoea. *Eimeria* oocysts were excreted by 90.2% (110/122) of the calves on at least one day, and were excreted by calves originating from all of the seven farms. This is not surprising, as *Eimeria* infections commonly occur in young cattle, and environmental contamination with *Eimeria* oocysts is high [15, 18, 66, 67]. In previous studies, individual animal-level *Eimeria* infection rates varied from 28 to 83.7%, while herd prevalences ranged between 86 and 100% [15–21]. Weber et al. [22] reported excretion rates of 36.7% for *Eimeria* spp. oocysts in Swiss calves aged 26–49 days. However, with respect to comparing our results with those of other studies, we need to evidence that the same calves were sampled repeatedly in the present study, which increased the probability of detecting positive calves. In the end, our study confirms that calves on Swiss fattening farms have high rates of excretion of *Eimeria* oocysts.

Overall, 64.8% (79/122) of the calves excreted *Eimeria* oocysts within the first 7 days after arrival at the farm. Excreted unsporulated oocysts of *Eimeria* are not directly infective, and sporulation may take from 2 days to up to several weeks, depending on the environmental conditions [23]. The prepatent period varies between 6 and 21 days, depending on the species of *Eimeria* [47]. This suggests that a substantial number of the animals found positive for *Eimeria* were infected already on the dairy farm of origin. Calves can, in fact, become infected shortly after birth. This was confirmed by Faber et al. [68] and Sánchez et al. [12], who were able to detect *Eimeria*-positive calves at a young age, from 15 to 21 days old. In opposition to the number of calves testing positive within the first 7 days, only 31 (25.4%, *n* = 122) tested positive for the first time on the last examination days (days 14 and 28). The results of the present study therefore indicate that most of the infected animals were already shedding oocysts at the regrouping stage. However, since some of the animals were excreting oocysts at the end of the study (day 28), reinfections may also have occurred on the fattening farms. As a consequence of the shedding of oocysts, the group pens on the fattening farms could have become contaminated shortly after arrival of the calves, which may have led to the infection of more calves, or the reinfection of calves. The results of the first examination days, after the arrival of the animals on the fattening farms, did not indicate that a metaphylactic treatment against coccidia of the entire group was necessary to reduce the risk of diarrhoea or to reduce the infection pressure. However, this should be considered in cases of clinical coccidiosis on fattening farms, based on the assumption that the implementation of appropriate management strategies also contribute to good calf health. The findings presented here provide support for the premise that prevention should begin at the dairy farm, to avoid severe clinical outbreaks on the fattening farm. Thus, one aim should be to keep stress due to transportation low and, contemporaneously, not completely eliminating the pathogen, as a low infection pressure promotes the development of appropriate immune responses.

The highest *Eimeria* OPG values were observed on the first 3 days of examination, days 1, 4 and 7, with the percentage of calves with an OPG above 5000 higher on these days than on days 14 and 28. This finding is consistent with the results of Bangoura et al. [15], who observed that animals had higher OPG values when they were tested earlier after rehousing compared to later. They hypothesised that their results were related to the stress caused to the animals when they were moved and regrouped from different farms.

There were higher odds for the calves to excrete more *Eimeria* OPG during the warm season. Previous studies have described the impact of climatic conditions on the prevalence of *Eimeria* [12, 14, 69], although they did not always clearly show which conditions favour higher infection rates. However, warm and wet weather was found to favour the development of oocysts in the environment and, thus, may increase the risk of infection [70]. In contrast, temperatures below 15 °C and humidity below 80% reduced the odds of clinical coccidiosis [14, 71]. Thus, the influence of specific geographic conditions on infection must also be considered [18].

In addition to quantitative evaluation, distinguishing infections with pathogenic from non-pathogenic *Eimeria* oocysts is important. Various morphological characteristics of the sporulated oocysts are used as the gold standard for *Eimeria* differentiation [72]. Here, with the animals kept in stalls, it was clinically relevant to differentiate the most pathogenic species, *E. zuernii* and *E. bovis*, as *E. alabamensis* only causes problems in calves kept on pasture [27]. Therefore, we measured and identified 50 non-sporulated oocysts from each positive sample and classified them as *E. zuernii*, *E. bovis*, and apathogenic *Eimeria* spp. To overcome size overlap, the size ranges of *E. zuernii* and *E. bovis* were approximated to their mean values. This procedure can result in a slight underestimation of pathogenic species, but allows an efficient and immediately available means of differentiating species of relevance.

It has been suggested that younger calves excrete higher numbers of *Eimeria* oocysts [12, 15]. In the present study, a correlation between age and OPG was identified only for *E. zuernii*, with calves having higher OPGs when they were younger compared to calves on examination day 28. This correlation may partly be explained by the fact that calves, once infected, develop a good immune response against the pathogenic species *E. bovis* and *E. zuernii* [14]. Our study also showed that *E. zuernii* was less common than *E. bovis:* therefore, younger animals may not have been exposed to *E. zuernii* on the farm of origin, implying that they did not have an immune response or that it was inadequate. The young calves may have excreted a higher numbers of *E. zuernii* oocysts compared to when they were older due to a combination of stress and a potentially higher infection pressure on the fattening farms.

*Giardia* cysts were detected on all of the investigated farms, with an overall excretion rate of 73%. Higher infection rates were found in the present study compared to those reported by Weber et al. [22], who found a prevalence of 35% in Swiss calves aged 26–49 days, and those found in other European countries (France 39.6%, Germany 51.2%, Italy 32.2%, the UK 54.9%) [31]. However, the fact that all of the animals in the present study were examined on five occasions increased the chance of detecting *Giardia*. Possibly as a result of carrying out repeated testing, we observed that *Giardia* infections occurred more frequently in the cold (80.3%, 53/66) than in the warm (64.3%, 36/56) season, which is comparable to the results of previous studies [31, 33, 73]. In contrast, Wade et al. [74] found that *Giardia* sp. infections were more common in calves in the summer, and Xiao et al. [75] reported a higher risk in spring. A possible explanation for the peak of *Giardia* excretion found in the present study during winter months is that more calves suffer from serious respiratory diseases then, so may also be more susceptible to *Giardia* infections in the winter. Alternatively, specific geographical locations and favourable environmental conditions may lead to a higher infection rate and pressure [33, 76, 77].

Although *G. duodenalis* is found worldwide [78], the relevance of infections with this parasite in production animals has not yet been fully elucidated [73]. In our study, *Giardia* positivity was not associated with clinical diarrhoea. *Giardia duodenalis* may cause diarrhoea and affect animal performance [38, 79], but subclinical infections are more common [31, 32]. Also, *Giardia* infections may be of relevance to public health if calves are infected with zoonotic assemblages. However, this is presently of minor concern as cattle are usually infected with the non-zoonotic assemblage E [80]. In contrast to other studies on calves [52, 81], no potentially zoonotic *Giardia* assemblage was isolated from the calves in our study. Lichtmannsperger et al. [82] identified a single sample containing zoonotic *Giardia* assemblage A out of 177 examined faecal samples from dairy calves younger than 180 days with diarrhoea, which they investigated in neighbouring Austria.

*Cryptosporidium* was found on five out of the seven farms, but the positivity of the animals was not associated with clinical diarrhoea. Despite repeated testing, we observed a low excretion rate, 11.5%, in contrast to that reported by Weber et al. [22], who reported a prevalence of 33.3% in Swiss calves at the age of 26–49 days. In the present study the mean age of the calves was 37.3 days (SD = 10.3). Previous studies, including Lichtmannsperger et al.’s [82], showed that *Cryptosporidium* is most common, with a significantly higher intensity of oocyst shedding, in animals aged 1–2 weeks. Due to the zoonotic potential of *C. parvum*, and the presence of *C. parvum* in humans and other animals [38, 40], further tests are needed to assess the potential risk of this parasite to humans. *Cryptosporidium bovis* and *C. ryanae* were also identified in the faecal samples; however, similar to previous studies [50, 83, 84], infection rates with these species were low. *Cryptosporidium bovis*, *C. ryanae* and *C. andersoni* are all of low clinical relevance [85].

During the study period, half of the calves suffered from diarrhoea, independently of season. The calves most frequently had diarrhoea on day 4 and day 7 after arrival at the farms. On those days, the calves were between 25 and 64 days old (mean = 42.1, SD = 10.0). Koutny et al. [18] showed that diarrhoea was most common in calves between 14 and 28 days of age. The animals in our study were older than this, but in their review, Swanson and Morrow-Tesch [86] concluded that stress due to transport, in addition to many other factors, can lead to morbidity, which may explain the occurrence of diarrhoea in the calves also at a later stage.

Interestingly, there was no correlation between the excretion of protozoal pathogens and the occurrence of diarrhoea. However, since the calves were only clinically assessed at the time of sample collection, it cannot be excluded that the pathogen-positive calves suffered from diarrhoea between the examinations. Stress-induced disruption of intestinal integrity is believed to play an important role in the host–pathogen interaction in *Eimeria* infections. It is hypothesised that supporting gut health is an important measure in protecting calves from clinical disease. The diarrhoea observed in the present study was predominantly mild and not bloody, so that treatment was not considered necessary. One animal was suffering from haemorrhagic diarrhoea, combined with reduced general condition. Faecal examination showed that it had a high *Eimeria* OPG (35,100), with *E. zuernii* oocysts prevailing. This calf was therefore treated with diclazuril (Vecoxan, oral suspension, 1 mg/kg body weight) and its condition subsequently improved within 2 days. While several studies showed a correlation between the excretion of *Eimeria* oocysts and the onset of diarrhoea, especially in association with *E. zuernii* [15, 16, 24, 67], other studies showed that OPG values did not necessarily correlate to the severity of clinical illness [19, 66, 70]. In our study, animals frequently exhibited high OPGs [e.g. the highest OPGs on days 1, 4 and 7 in individual calves were 71,000, 108,400 and 40,200, respectively (see Additional file 4: Table S4], without suffering from diarrhoea at the same time. Subclinical infections were thus frequently present, as reported by Mitchell et al. [69]; however, prepatency and other possible inconsistencies between the clinical picture and OPG values need to be considered.

One of the selection criteria for the farms in our study was no history of clinical coccidiosis, as our aim was to investigate the dynamics and the presence or absence of protozoal pathogens on representative farms that had not had any problems of this nature. This criterion could obviously have been a confounder. Thus, an additional step could be to adopt an identical sampling scheme for farms with animals that have had diarrhoea, and then compare the dynamics of infection between the two types of farm. Moreover, as diarrhoea is a complex, multifactorial sign induced by infectious and non-infectious factors, even if protozoal co-infections had been considered in our models, other agents such as bacterial or viral pathogens and factors such as diet, environmental conditions, management, and immune status could have had an influence on faecal consistency [8, 87]. On the other hand, the host also plays an important role in the host–pathogen interaction. The intestinal epithelial cells act as a barrier to infection [88], and animals with good gut health can deal better with an infection than animals with poor intestinal health. There are many factors, such as weaning, heat stress and ruminal acidosis, which negatively affect the integrity of the intestine, and compromising the integrity of the intestinal barrier may lead to a condition known as leaky gut [89, 90]. Thus, factors other than protozoa may play a more important role in the onset of diarrhoea after the arrival of calves at a fattening farm, as the animals are confronted with various stressors at this time.

## Conclusions

Many factors may influence the health and body weight gain of calves at a fattening farm. Although several animals in the present study had diarrhoea in the first 14 days after arrival at the fattening farms, no association between the presence of protozoal pathogens and diarrhoea or body weight gain was found. The excretion rates of *Eimeria* and *Giardia* in the Swiss fattening calves, which were aged 19–98 days, were high during the first 28 days on the fattening farms. *Eimeria* excretion occurred mainly in the warm season, while *Giardia* was mainly found in the cold season, confirming that both of these protozoal genera are subject to seasonality.

Even though correlations with diarrhoea were not found in this study, we should consider the effects of subclinical infections. Remarkably, some of the calves arrived at the fattening farm with an established *Eimeria* infection. Thus, preventive measures must be applied on farms of origin to keep infection pressure on fattening farms as low as possible. The identification of patterns of excretion of intestinal protozoa can help us to better understand the dynamics of infections with potentially relevant pathogens. This is of value for the development of preventive measures for application at the most appropriate time with the aim of improving the health of calves at a critical phase of their lives.

### Supplementary Information

**Additional file 1: Figure S1.** Relative proportions of *Eimeria-*negative and *Eimeria-*positive calves [divided into categories according to low (< 500), intermediate (500–5000) or high (> 5000) numbers of oocysts per gram (OPG) of faeces] on seven farms sampled during the warm (*n* = 3) or the cold (*n* = 4) seasons on days 1, 4, 7, 14 and 28 after arrival and grouping of the animals at the fattening farms.

**Additional file 2: Figure S2.** Relative proportions of the pathogenic species *Eimeria zuernii* and *Eimeria bovis,* as well as the not further differentiated apathogenic *Eimeria *spp., in 122 calves examined on days 1, 4, 7 and 28 after arrival at the fattening farm.

**Additional file 3: Table S1.** Number of calves positive for *Eimeria* (all species), *Eimeria zuernii*, *Eimeria bovis*, *Giardia* and *Cryptosporidium* over the entire study period on each farm. *Eimeria *results are presented in more detail: number of calves that excreted > 5000 OPG of faeces at least once, and the number of animals that tested positive for *Eimeria* before and after day 14, respectively.

**Additional file 4: Table S2.** Presence of diarrhoea, and protozoal pathogens in faecal samples, of calves examined during the cold season on days 1, 4, 7, 14, 28 after arrival at the fattening farm [positive sample (+) vs. empty field (not detected)]. Farm number, animal ID, body weight on day 1 and 28 (and, accordingly, body weight gain), and age at day 1. *D* Diarrhoea,* E* *Eimeria*,* Z* *Eimeria zuernii*,* B* *Eimeria bovis*,* G* *Giardia*,* C* *Cryptosporidium*. **Table S3.** Presence of protozoal pathogens and diarrhoea in faecal samples of calves examined during the warm season on examination days 1, 4, 7, 14, 28 after arrival at the fattening farm [positive sample (+) vs. empty field (not detected)]. Farm number, animal ID, body weight on days 1 and 18 (and, accordingly, body weight gain) and age at day 1. *D* Diarrhoea,* E* *Eimeria*,* Z* *Eimeria zuernii*,* B* *Eimeria bovis*,* G* *Giardia*,* C* *Cryptosporidium*. **Table S4.** All of the data.

## Data Availability

All the relevant data are included herein. Remaining material used for the biomolecular analyses, and partially frozen faecal samples, are available on request.
