# Supplementary figures and images for: Dynamics of protozoal excretion in the faeces of calves during the first 28 days after arrival at the fattening farm indicate infection before regrouping and show poor temporal correlation with diarrhoea
Source: Parasit Vectors. 2023 Sep 27;16:338. doi: 10.1186/s13071-023-05911-0 (PMC10523781; doi:10.1186/s13071-023-05911-0)

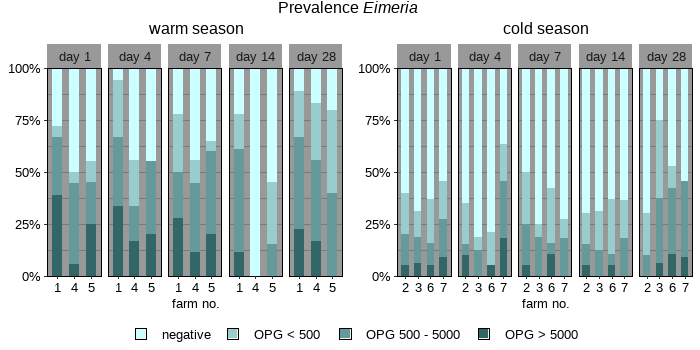

Supplement: Supplementary file 1 — Additional file 1: Figure S1. Relative proportions of Eimeria-negative and Eimeria-positive calves [divided into categories according to low (< 500), intermediate (500–5000) or high (> 5000) numbers of oocysts per gram (OPG) of faeces] on seven farms sampled during the warm (n = 3) or the cold (n = 4) seasons on days 1, 4, 7, 14 and 28 after arrival and grouping of the animals at the fattening farms. [file 13071_2023_5911_MOESM1_ESM.tiff]

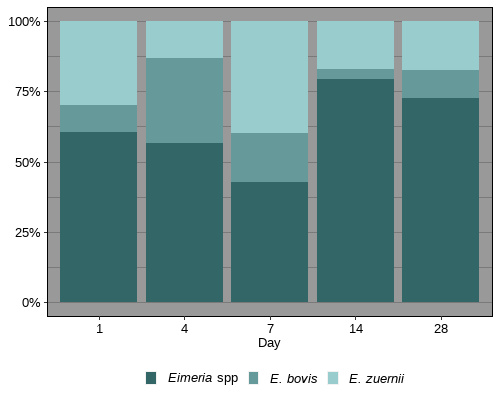

Supplement: Supplementary file 2 — Additional file 2: Figure S2. Relative proportions of the pathogenic species Eimeria zuernii and Eimeria bovis, as well as the not further differentiated apathogenic Eimeria spp., in 122 calves examined on days 1, 4, 7 and 28 after arrival at the fattening farm. [file 13071_2023_5911_MOESM2_ESM.tiff]
