# Supplementary material for: Dynamics of protozoal excretion in the faeces of calves during the first 28 days after arrival at the fattening farm indicate infection before regrouping and show poor temporal correlation with diarrhoea
Source: Parasit Vectors. 2023 Sep 27;16:338. doi: 10.1186/s13071-023-05911-0 (PMC10523781; doi:10.1186/s13071-023-05911-0)
Supplement: Supplementary file 3 — Additional file 3: Table S1. Number of calves positive for Eimeria (all species), Eimeria zuernii, Eimeria bovis, Giardia and Cryptosporidium over the entire study period on each farm. Eimeria results are presented in more detail: number of calves that excreted > 5000 OPG of faeces at least once, and the number of animals that tested positive for Eimeria before and after day 14, respectively. [file 13071_2023_5911_MOESM3_ESM.docx]

Table S1: Number of calves positive for *Eimeria* (all species), *E. zuernii*, *E. bovis*, *Giardia* and *Cryptosporidium* over the entire study period on each farm. *Eimeria* results are presented more in detail: number of calves that at least once excreted more than 5000 oocysts per gram of faeces and the number of animals that tested positive for *Eimeria* before and after day 14, respectively. Farm 7= 11.

|  | **Farm 1 (n=18)** | | **Farm 2 (n=20)** | | **Farm 3 (n=16)** | | **Farm 4 (n=18)** | | **Farm 5 (n=20)** | | **Farm 6 (n=19)** | | **Farm 7 (n=11)** | |
| --- | --- | --- | --- | --- | --- | --- | --- | --- | --- | --- | --- | --- | --- | --- |
|  | N | % | N | % | N | % | N | % | N | % | N | % | N | % |
| *Eimeria* | | | | | | | | | | | | | | |
| positive | 17 | 94.4 | 17 | 85.0 | 14 | 87.5 | 18 | 100.0 | 20 | 100.0 | 15 | 78.9 | 9 | 81.8 |
| > 5000 OPG | 16 | 88.9 | 3 | 15.0 | 2 | 12.5 | 8 | 44.4 | 8 | 40.0 | 4 | 21.1 | 4 | 36.4 |
| positive < day 14 | 17 | 94.4 | 13 | 65.0 | 6 | 37.5 | 11 | 61.1 | 14 | 70.0 | 11 | 57.9 | 7 | 63.6 |
| positive > day 14 | 0 | 0.0 | 4 | 20.0 | 8 | 50 | 7 | 38.9 | 6 | 30.0 | 4 | 21.1 | 2 | 18.2 |
| *E. zuernii* | | | | | | | | | | | | | | |
| positive | 16 | 88.9 | 5 | 25.0 | 5 | 31.25 | 8 | 44.4 | 7 | 35.0 | 7 | 36.8 | 3 | 27.3 |
| > 5000 OPG | 3 | 16.7 | 0 | 0.0 | 0 | 0 | 0 | 0.0 | 1 | 5.0 | 1 | 5.3 | 1 | 9.1 |
| positive < day 14 | 13 | 72.2 | 4 | 20.0 | 3 | 18.75 | 8 | 44.4 | 6 | 30.0 | 1 | 5.3 | 1 | 9.1 |
| positive > day 14 | 3 | 16.7 | 1 | 5.0 | 2 | 12.5 | 0 | 0.0 | 1 | 5.0 | 6 | 31.6 | 2 | 18.2 |
| *E. bovis* | | | | | | | | | | | | | | |
| positive | 16 | 88.9 | 10 | 50.0 | 7 | 43.75 | 10 | 55.6 | 13 | 65.0 | 7 | 36.8 | 5 | 45.5 |
| > 5000 OPG | 8 | 44.4 | 0 | 0.0 | 0 | 0 | 3 | 16.7 | 4 | 20.0 | 1 | 5.3 | 0 | 0.0 |
| positive < day 14 | 13 | 72.2 | 6 | 33.3 | 5 | 31.25 | 7 | 38.9 | 11 | 55.0 | 3 | 15.8 | 4 | 36.4 |
| positive > day 14 | 3 | 16.7 | 4 | 20.0 | 2 | 12.5 | 3 | 16.7 | 2 | 10.0 | 4 | 21.1 | 1 | 9.1 |
| *Giardia* | | | | | | | | | | | | | | |
| positive | 9 | 50.0 | 15 | 75.0 | 14 | 87.5 | 14 | 77.8 | 13 | 65.0 | 14 | 73.7 | 10 | 90.9 |
| *Cryptosporidium* | | | | | | | | | | | | | | |
| positive | 5 | 27.8 | 0 | 0.0 | 3 | 18.8 | 1 | 5.6 | 4 | 20.0 | 0 | 0.0 | 1 | 9.1 |
